# Application of the Inverse-Electron-Demand Diels-Alder Reaction for Metabolic Glycoengineering

**DOI:** 10.3389/fchem.2021.654932

**Published:** 2021-04-13

**Authors:** Lisa Maria Haiber, Markus Kufleitner, Valentin Wittmann

**Affiliations:** Department of Chemistry and Konstanz Research School Chemical Biology (KoRS-CB), University of Konstanz, Konstanz, Germany

**Keywords:** inverse-electron-demand Diels-Alder reaction, carbohydrates, metabolic engineering, bioorthogonal chemistry, tetrazines

## Abstract

The inverse electron-demand Diels-Alder (IEDDA or DA_inv_) reaction is an emerging bioorthogonal ligation reaction that finds application in all areas of chemistry and chemical biology. In this review we highlight its application in metabolic glycoengineering (MGE). MGE is a versatile tool to introduce unnatural sugar derivatives that are modified with a chemical reporter group into cellular glycans. The IEDDA reaction can then be used to modify the chemical reporter group allowing, for instance, the visualization or isolation of glycoconjugates. During the last years, many different sugar derivatives as well as reporter groups have been published. These probes are summarized, and their chemical and biological properties are discussed. Furthermore, we discuss examples of MGE and subsequent IEDDA reaction that highlight its suitability for application within living systems.

## Introduction

The Diels-Alder reaction (Diels and Alder, 1928) is widely used for stereoselective synthesis of complex molecules, such as many natural products or pharmaceuticals (Brieger and Bennett, 1980; Nicolaou et al., 2002; Funel and Abele, 2013). In 1959, a variant thereof, the inverse-electron-demand Diels-Alder (IEDDA or DA_inv_) reaction of 1,2,4,5-tetrazines and electron-rich dienophiles was reported for the first time (Carboni and Lindsey, 1959). However, it took until 2008 when the IEDDA reaction between 1,2,4,5-tetrazines and trans-cyclooctenes (Blackman et al., 2008), cyclobutenes (Braun et al., 2008), or norbornenes (Devaraj et al., 2008) was applied in bioconjugation. The rate constants of the IEDDA reaction can span a range of many orders of magnitude depending on the used tetrazine and dienophile structure, and it can be performed in aqueous media which even accelerates the reaction (Wijnen et al., 1996). Among the dienophiles with fastest reaction kinetics are trans-cyclooctene (TCO) derivatives with a three-membered ring fused to the eight-membered cyclooctene (sTCO) resulting in second-order rate constants up to 10^6^ M^−1^ s^−1^ (Taylor et al., 2011).

Although norbornene and especially TCO derivatives react rapidly in the IEDDA reaction even exceeding the kinetics of well-established bioorthogonal ligation reactions, such as the copper-catalyzed azide-alkyne [3 + 2] cycloaddition (CuAAC) (Rostovtsev et al., 2002; Tornøe et al., 2002), their size limits potential applications as reporter groups for metabolic labeling. From extensive studies of Sauer and coworkers it was known that 3-methyl-cyclopropene reacts rapidly with tetrazines (Thalhammer et al., 1990). However, cyclopropenes are prone to polymerization and were known to be unstable toward thiols and other potential nucleophiles present in a cell preventing their use in cellular systems. To overcome these limitations, the Deveraj (Yang et al., 2012) and Prescher (Patterson et al., 2012) groups investigated the effects of substituents on cyclopropene stability and reactivity. Methyl substituents at the double bond greatly increased the stability of the cyclopropenes toward nucleophiles as well as polymerization leading to the first dienophile-modified carbohydrate derivative suitable for metabolic labeling (Patterson et al., 2012).

The IEDDA reaction is not only fast but also chemoselective and irreversible due to a subsequent retro-Diels-Alder reaction under nitrogen release. Furthermore, it does not require toxic heavy metal catalysts. These features make the IEDDA reaction an excellent choice for numerous applications as bioorthogonal ligation reaction in all areas of chemistry and chemical biology (Knall and Slugovc, 2013; Wu and Devaraj, 2016; Mayer and Lang, 2017; Oliveira et al., 2017). One of these applications is metabolic glycoengineering (Kayser et al., 1992; Mahal et al., 1997; Palaniappan and Bertozzi, 2016; Sminia et al., 2016; Wratil et al., 2016). The pivotal roles that carbohydrates play in biology, for example during protein regulation, cell adhesion, or immune response, led to various approaches to study their biological functions. In metabolic glycoengineering (MGE), cells are incubated with synthetic carbohydrate derivatives which are equipped with an unnatural functional group, named a chemical reporter group (Prescher and Bertozzi, 2005). The sugars are metabolized by the enzymatic machinery of the cell and are incorporated into glycan structures in competition to their natural equivalents. Subsequent modification of the reporter group can be achieved by a bioorthogonal ligation reaction (Sletten and Bertozzi, 2009) to allow, for example, the visualization of glycosylation during different cellular conditions in health and disease, the isolation of glycoconjugates, or targeted delivery strategies (Wang et al., 2017). To facilitate cellular uptake by passive diffusion, the carbohydrate derivatives are usually employed in their O-acetylated form. Inside cells, O-deacetylation is catalyzed by non-specific esterases. The effect of added sugars on the carbohydrate metabolism of the cell is a topic of current investigations (Dold and Wittmann, 2021).

Multiple sugar analogues with different reporter groups have been synthesized over the years including a ketone-modified mannosamine derivative (Mahal et al., 1997), azido sugars (Saxon and Bertozzi, 2000), alkyne sugars (Hsu et al., 2007), isonitrile-modified sugars (Stairs et al., 2013), and others summarized in the above-mentioned review articles. More recently, dienophile-modified sugars that can be reacted in a IEDDA reaction have been added to the MGE toolbox, and they are the topic of this review. In the following section, we will first present a chronological order of dienophile-modified carbohydrates before we will highlight some applications of these derivatives.

## Chronology of Dienophile-Modified Carbohydrates

**FIGURE 1:**
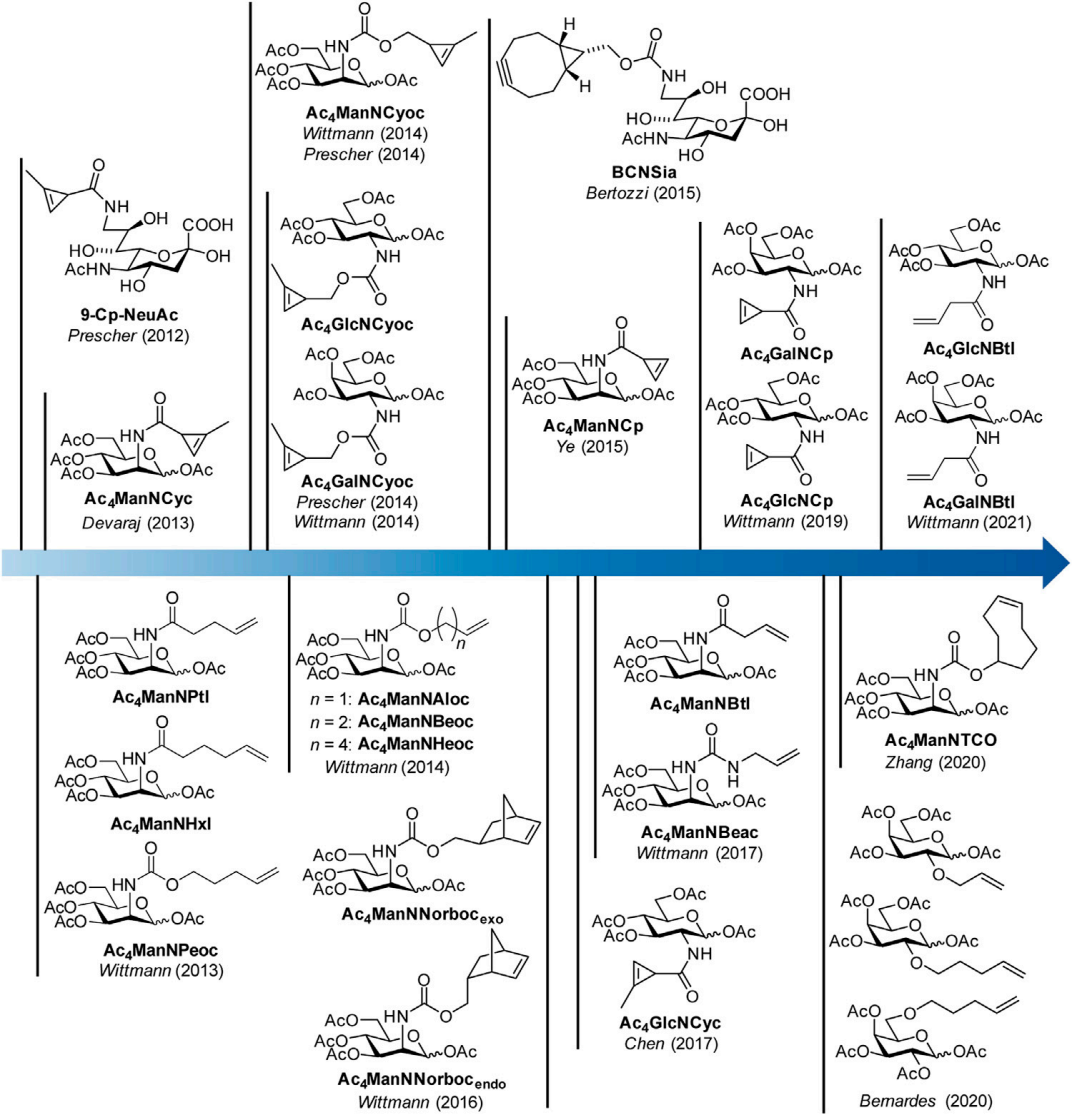
Timeline of synthetic carbohydrate derivatives for MGE with dienophile reporter groups for bioorthogonal labeling with the IEDDA reaction.

In 2012, the Prescher group reported the first example of a dienophile-modified carbohydrate, a sialic acid with a methyl-substituted cyclopropene linked by an amide bond to the 9-position (9-Cp-NeuAc) (Patterson et al., 2012) (Figure 1). 9-Cp-NeuAc was incorporated into Jurkat cell surface glycans and labeled in a two-step process with a tetrazine-biotin and a streptavidin-dye conjugate. Successful incorporation was quantified with flow cytometry. Additionally, the concurrent utilization of methylcyclopropene and azide reporters for dual labeling was shown. In the following year, our group introduced terminal alkene-modified mannosamine derivatives with different chain length and an amide (Ac_4_ManNPtl and Ac_4_ManNHxl) or a carbamate linkage (Ac_4_ManNPeoc) (Niederwieser et al., 2013). MGE experiments and confocal fluorescence microscopy revealed their metabolic acceptance and incorporation into glycans on the surface of HEK 293T and HeLa S3 cells. The combination of IEDDA and click chemistry allowed for the first time the detection of two different carbohydrates–one modified with a terminal alkene, another with an azide—within one experiment. In the following, a variety of mannosamines derivatized with carbamate-linked terminal alkenes (Ac_4_ManNAloc, Ac_4_ManNBeoc, Ac_4_ManNHeoc) were synthesized and compared unraveling the correlation between staining intensity on the one hand and metabolic acceptance and reaction rate depending on the chain length on the other hand (Späte et al., 2014c). In addition, glucosamine analogues with carbamate-linked terminal alkenes were reported. However, cell experiments showed that these sugars are cell toxic at the concentrations necessary for MGE.

Also in 2013, the Devaraj group introduced the mannosamine analogue Ac_4_ManNCyc modified with the same amide-linked methylcyclopropene used by Prescher (Cole et al., 2013). They could show that the sugar is processed by different human cancer cell lines and displaced on the cell surface allowing visualization by confocal fluorescence microscopy after IEDDA labeling with a tetrazine-dye conjugate. Based on the observation of the Devaraj group (Yang et al., 2012) that carbamate-linked methylcyclopropenes react about 100 times faster with tetrazines than amide-linked ones, we (Späte et al., 2014a) and later on the Prescher group (Patterson et al., 2014) reported the mannosamine derivative Ac_4_ManNCyoc with a carbamate-linked cyclopropene. The higher IEDDA reactivity of the carbamate-linked cyclopropene significantly reduced the time needed for the labeling step compared to the amide-linked cyclopropene enabling an efficient one-step labeling approach with a tetrazine-dye conjugate. This improved methylcyclopropene reporter was also used to modify glucosamine (Ac_4_GlcNCyoc) as well as galactosamine (Ac_4_GlcNCyoc) (Späte et al., 2014b; Patterson et al., 2014).

In 2015, a bicyclononyne-modified sialic acid (BCNSia) was synthesized and incorporated into the glycans of various cell lines (Agarwal et al., 2015). Additionally, it was used to investigate sialylation in zebra fish embryos. In the same year, mannosamine with a minimal amide-linked cyclopropene reporter, Ac_4_ManNCp, was published by the Ye group (Xiong et al., 2015). This derivative appeared to be surprisingly stable although it lacks the methyl substituent and reacted 9-fold faster with a tetrazine than Ac_4_ManNCyc. In a direct comparison, the new sugar gave higher fluorescence intensities after IEDDA labeling than the amide-linked methylcyclopropene in MGE experiments in several cell lines.

In the following years, norbornene-modified mannosamine derivatives (Ac_4_ManNNorboc_endo/exo_) (Späte et al., 2016), a shorter amide-linked terminal alkene (Ac_4_ManNBtl) and an urea-linked terminal alkene (Ac_4_ManNBeac) (Dold et al., 2017) extended the pool of reporter groups for the IEDDA reaction. Additionally, the glucosamine derivative Ac_4_GlcNCyc of the amide-linked methylcyclopropene was synthetized (Zhu and Chen, 2017). In 2019, glucosamine and galactosamine derivatives of the unsubstituted amide-linked cyclopropene (Ac_4_GlcNCp and Ac_4_GalNCp) complemented the cyclopropene series (Hassenrück and Wittmann, 2019). Only recently, the use of galactose analogues modified with terminal alkenes (Kitowski and Bernardes, 2020), a TCO-modified mannosamine derivative (Ac_4_ManNTCO) (Zhang et al., 2020) and the butenoyl derivatives Ac_4_GlcNBtl and Ac_4_GalNBtl (Dold and Wittmann, 2021) were reported.

## Reporter Groups

### Cyclopropene Reporters

Cyclopropene reporters, that have been developed for MGE, comprise an amide-linked methylcyclopropene (Cyc), a carbamate-linked methylcyclopropene (Cyoc), and a minimal amide-linked cyclopropene (Cp). In the following, the mannosamine derivatives of these three cyclopropenes are compared (Hassenrück and Wittmann, 2019). The minimal amide-linked cyclopropene (Ac_4_ManNCp) has a higher IEDDA reactivity than the amide-linked methylcyclopropene (Ac_4_ManNCyc) and also a higher incorporation efficiency (IE)^*^ in HEK 293T cells (Table 1) resulting in a brighter cell surface staining as determined by flow cytometry and confocal fluorescence microscopy. The lack of the methyl group clearly improves the reporter. Comparison of the methylcyclopropenes Ac_4_ManNCyc and Ac_4_ManNCyoc shows that the carbamate linkage increases the reactivity in the IEDDA reaction, while it significantly decreases the IE. In the end, cell surface staining was more intense for Ac_4_ManNCyoc. In this case, the higher reactivity of ManNCyoc outweighs the lower IE. For Ac_4_ManNCyoc and Ac_4_ManNCp, the observed cell surface staining is nearly the same. However, the smaller Ac_4_ManNCp is better accepted than Ac_4_ManNCyoc leading to a higher IE for the amide-linked minimal cyclopropene. The reaction kinetics behave vice versa. Here, the higher IE of ManNCp and the higher reactivity of ManNCyoc balance each other out.

**TABLE 1:** Incorporation efficiencies (IE) as sialic acids in MGE experiments and second-order rate constants *k*_2_ of aminosugar derivatives with dienophile reporter groups (Niederwieser et al., 2013; Späte et al., 2014c; Späte et al., 2016; Dold et al., 2017; Hassenrück and Wittmann, 2019; Dold and Wittmann, 2021).

		Amide derivatives			Carbamate derivatives			Urea derivative		
	Chain length^a^		IE [%]	*k*_2_ [m ^−1^s^−1^]^b^		IE [%]	*k*_2_ [m ^−1^s^−1^]^b^		IE [%]	*k*_2_ [m ^−1^s^−1^]^b^
Cyclopropenes		Ac_4_ManNCyc	50^c^	0.03	Ac_4_ManNCyoc	4.9^c^	1.0			
		Ac_4_ManNCp	72^c^	0.09						
		Ac_4_GlcNCp	3.5^c^		Ac_4_GlcNCyoc	Not detected^c^				
Terminal alkenes	4	Ac_4_ManNBtl	62	0.0011						
	5	Ac_4_ManNPtl	31	0.021	Ac_4_ManNAloc	50	0.0015			
	6	Ac_4_ManNHxl	8.3	0.041	Ac_4_ManNBeoc	15	0.014	Ac_4_ManNBeac	6.6	0.029
	7				Ac_4_ManNPeoc	3.7	0.038			
	8				Ac_4_ManNHeoc	0.3	0.074			
	4	Ac_4_GlcNBtl	Not detected							
	4	Ac_4_GalNBtl	Not detected							
Norbornenes					Ac_4_ManNNorboc_exo_	≈1	4.6			
					Ac_4_ManNNorboc_endo_	≈1	2.0			

^a^ Length of the acyl side chain including the carbonyl C atom.

^b^ Second-order rate constants were determined in all cases for reaction of the water-soluble deacetylated mannosamine derivatives with a water-soluble 3-phenyl-6-(pyrimidin-2-yl)-1,2,4,5-tetrazine in acetate buffer (pH 4.8).

^c^ Determined with the corresponding cyclopropane derivatives.

The Cyoc reporter was also attached to glucosamine and galactosamine and the resulting derivatives Ac_4_GlcNCyoc and Ac_4_GalNCyoc used for MGE. When Jurkat cell lysate was analyzed by Western blot, Ac_4_GlcNCyoc resulted in a more intense labeling than the corresponding azide derivative whereas Ac_4_ManNCyoc and Ac_4_GalNCyoc gave a similar intensity compared to the corresponding azide derivatives (Patterson et al., 2014). The three Cyoc derivatives were also used for MGE with HeLa S3 cells (Späte et al., 2014b). Western blot analysis of cell lysate resulted in a significantly stronger staining intensity for cells that had been cultivated with Ac_4_GlcNCyoc. Since the sample preparation included the fraction of intracellular proteins and O-GlcNAcylation is a modification primarily found for cytosolic and nuclear proteins, it was suggested that Ac_4_GlcNCyoc is suitable to target O-GlcNAcylated proteins. Analysis of cell-surface staining of HEK 293T and HeLa S3 cells by confocal microscopy on the other hand revealed that Ac_4_ManNCyoc induced the most intense staining of the three Cyoc derivatives.

When the Cp and Cyoc derivatives of glucosamine were compared in HEK 293T cells, Ac_4_GlcNCp showed a much brighter cell-surface staining than Ac_4_GlcNCyoc whereas Ac_4_GlcNCyoc resulted in a more intense labeling in a Western blot analysis of cell lysate (Hassenrück and Wittmann, 2019). DMB-labeling experiments with the corresponding cyclopropane derivatives suggest that GlcNCp is converted into the corresponding sialic acid whereas GlcNCyoc is not.

Ac_4_GlcNCyoc was successfully applied for the visualization of protein-specific O-GlcNAcylation inside living cells (Doll et al., 2016; Doll et al., 2018). Proteins of interest were tagged with GFP. After MGE and labeling with a cell permeable tetrazine-TAMRA conjugate, O-GlcNAcylation of the protein of interest as well as its localization within the cell was detected by FLIM-FRET microscopy. This example highlights the advantage of the IEDDA chemistry of not requiring a toxic catalyst enabling its application within a living cell.

Ac_4_GlcNCyoc was also used to image glycans in Arabidopsis roots (Hoogenboom et al., 2016). In comparison to CuAAC, the IEDDA reaction showed a more uniform fluorescence signal with less background staining. The handling of the IEDDA reagents was easier and the redundant need for copper did not damage the cell wall of the plants. Additionally, also Ac_4_GlcNCyc (named Ac_4_GlcNCp in this publication) was used in *Arabidopsis thaliana* to evaluate the reporter performance for visualizing root carbohydrate structures by fluorescence imaging (Zhu and Chen, 2017). The observation that the carbamate-linked methylcyclopropene Cyoc has a low reactivity toward nitrile imines that are photochemically generated during the photoclick reaction, allowed the use of Ac_4_ManNCyoc in combination with azido- and acryl-mannosamine derivatives in a triple-orthogonal labeling approach (Schart et al., 2019).

### Terminal Alkene Reporters

Terminal alkenes were developed in parallel to cyclopropene reporters (Niederwieser et al., 2013) and extend the repertoire of reporter groups suitable for MGE. They are small, robust, and hardly found in biological systems. In proteins, they are completely absent. Both, their reactivity in the IEDDA reaction and metabolic acceptance are dependent on the chain length. Comparative studies with mannosamine derivatives (Dold et al., 2017) showed that shorter chain lengths are better accepted by the enzymes of the sialic acid biosynthesis. Longer chain lengths between the terminal alkene and the electron-withdrawing carbonyl group on the other hand increase the electron density of the double bond and, therefore, their reactivity in the IEDDA reaction. These two opposite effects lead to the observation that within a series of compounds a certain chain length represents the best balance between the two effects resulting in the most intense cell surface staining (Späte et al., 2014c).

Over the time, a pool of various terminal alkenes was developed (Niederwieser et al., 2013; Späte et al., 2014c; Dold et al., 2017; Dold and Wittmann, 2021). The compounds differed not only in their length of the side chain but also in the type of linkage to the amino sugar (amide, carbamate, urea). Table 1 gives an overview on the metabolic acceptance (in terms of IE values) and the second-order rate constants *k*_2_ of investigated compounds. Both within the series of amide derivatives and carbamate derivatives the two opposite effects on metabolic acceptance and reactivity mentioned above can be seen. Interesting is a comparison of three compounds that share the same length of the side chain but differ in the type of linkage: amide-linked Ac_4_ManNHxl, carbamate-linked Ac_4_ManNBeoc, and urea-linked Ac_4_ManNBeac. The carbamate is best accepted (IE = 15%), followed by the amide (IE = 8.3%) and the urea motif (IE = 6.6%). The order of reactivity is different with *k*_2_ values of 0.041 m ^−1^ s^−1^ for the amide, 0.029 m ^−1^ s^−1^ for the urea, and 0.014 m ^−1^ s^−1^ for the carbamate. For the cell-surface staining intensity observed by confocal fluorescence microscopy, the balance of IE and reaction kinetics is crucial. Using HEK 293T cells, the brightest staining was observed for amide-linked Ac_4_ManNHxl. Carbamate-linked Ac_4_ManNBeoc showed a weak staining whereas the urea-linked Ac_4_ManNBeac showed barely any staining (Dold et al., 2017).

Only recently, terminal-alkene derivatives of galactose were used for investigation of glycans in *Plasmodium*-infected hepatic cells (Kitowski and Bernardes, 2020). In this case the O2 or O6 position was modified by ether-linked terminal olefins of different chain length.

### Larger Ring-Strained Alkenes and Alkynes as Reporters

Ring-strained alkenes and alkynes, such as trans-cyclooctenes (TCO), bicyclononynes (BCN), or norbornenes, are among the fastest dienophiles for the IEDDA reaction (Lang et al., 2012). Unfortunately, their large size compared to other reporter groups can hamper the acceptance of unnatural sugar derivatives with these moieties by the enzymatic machinery (Jacobs et al., 2001; Pouilly et al., 2012; Dold et al., 2017). However, the following examples show that they are a viable alternative to cyclopropenes when reactivity and stability is more important than incorporation efficiency.

The first example is BCNSia, a sialic acid with a carbamate linkage to a BCN moiety at C-9 (Agarwal et al., 2015). This position was chosen for modification, since it is known that the enzymes involved in the incorporation of sialic acids tolerate even a larger alteration (Oetke et al., 2002; Han et al., 2005). BCN features a higher stability than cyclopropenes making it suitable for experiments with longer incubation times before further derivatization. BCNSia was employed to investigate sialylation in developing zebra fish embryos and besides known sialylation patterns new ones could be identified. Also this example highlights the benefit of the IEDDA reaction in living systems. With a tetrazine derivative that shows a strong increase in fluorescence after IEDDA reaction (Wu et al., 2014) it was possible to reduce background fluorescence.

Later, the potential of norbornene-modified mannosamine derivatives was explored (Späte et al., 2016). They react faster than cyclopropene derivatives with second order rate constants of 4.6 m ^−1^ s^−1^ for ManNNorboc_exo_ and 2.0 m ^−1^ s^−1^ for ManNNorboc_endo_. The differences in reaction rates between exo and endo derivatives, that had also been reported earlier (Vrabel et al., 2013), were observable when human cells were incubated with the unnatural sugars and labeled with a dye for fluorescence microscopy, flow cytometry, or Western blot analysis. The incorporation efficiency by DMB-labeling was determined to be about 1% for both derivatives. Additionally, the possibility for dual labeling together with click chemistry was shown for the norbornene derivatives (Späte et al., 2016) as well as BCNSia (Agarwal et al., 2015).

A TCO-modified mannosamine derivative (ManNTCO) was synthesized and used for MGE in human cancer cells and xenograft mice (Zhang et al., 2020). IEDDA reaction with a tetrazine-coated multi-spectral upconversion nanophosphor probe allowed to label the cell-surface glycans and monitor their level in living mice in real time. The probe can be applied as a replacement for common organic dyes with the potential to avoid tissue penetration and spontaneous fluorescence.

## Conclusion

The IEDDA reaction is a bioorthogonal ligation reaction that is now well-established for application in MGE. Many different carbohydrate reporters that differ in size and reactivity have been developed and chemically and biologically characterized. In combination with different tetrazines this represents a large toolbox that allows a fine tuning of a probe for the demands of different experiments. Whereas cyclopropene reporters feature high reactivity in combination with small size, terminal alkenes are chemically more stable enabling experiments with longer incubation times. The metabolic acceptance for probes that end up as sialic acids can be determined by DMB labeling. Depending on the size of the reporter group, the incorporation efficiency IE can vary greatly from below 1% up to more than 70%. This allows to select suitable probes for cases when the natural glycan structure should not be altered significantly or when an efficient incorporation is desired. Among the prime features of the IEDDA reaction is its compatibility with a living environment (cell, animal), because the reaction does not need toxic catalysts. This enables applications within living cells, such as the visualization of intracellular protein glycosylation in a spatially- and time-resolved manner. The option to combine the IEDDA reaction with other ligation reaction that can be orthogonal to the IEDDA reaction (click reaction, photoclick reaction) offers the option to perform dual- and even triple-labeling experiments. In this way, it becomes, for example, possible to quantify alterations of the levels of two or three carbohydrates (or other biomolecules) relative to each other in response to changing conditions. Its beneficial properties make the IEDDA reaction a valuable bioorthogonal ligation reaction not only for metabolic glycoengineering but for all sorts of applications in chemical biology.

## Footnotes

* The IE is the percentage of cellular sialic acids that is modified with the reporter group after MGE and can be determined by the DMB-labeling method (Dold et al., 2017). Since cyclopropenes are not stable under the acidic labeling conditions, their IE values have been approximated by using the corresponding cyclopropane derivatives during MGE (Hassenrück and Wittmann, 2019).
